# Morbidity, Including Fatal Morbidity, throughout Life in Men Entering Adult Life as Obese

**DOI:** 10.1371/journal.pone.0018546

**Published:** 2011-04-20

**Authors:** Esther Zimmermann, Claus Holst, Thorkild I. A. Sørensen

**Affiliations:** 1 Institute of Preventive Medicine, Copenhagen University Hospital, Copenhagen, Denmark; 2 Institute of Biomedical Sciences, University of Copenhagen, Copenhagen, Denmark; Universidad Peruana Cayetano Heredia, Peru

## Abstract

**Background:**

The association between obesity in adults and excess morbidity and mortality is well established, but the health impact throughout adult life of being obese in early adulthood needs elucidation. We investigated somatic morbidity, including fatal morbidity, throughout adulthood in men starting adult life as obese.

**Methods:**

Among 362,200 Danish young men, examined for military service between 1943 and 1977, all obese (defined as BMI≥31.0 kg/m^2^), and, as controls, a random 1% sample of the others was identified. In the age range of 18–25 years, there were 1,862 obese, which encompass the men above the 99.5 percentile, and 3,476 controls. Information on morbidity was obtained via national registers. Cox regression models were used to estimate the relative morbidity assessed as first incidence of disease, occurrence of disease in the year preceding death and prevalent disease at time of death.

**Results:**

From age 18 through 80 years the obese had an increased risk of becoming diseased by or die from a broad range of diseases. Generally, the incidence of first event, occurrence in the year prior to death, and prevalence at time of death showed the same pattern. As an example, the relative hazard of type 2 diabetes was constant throughout life at 4.9 (95% confidence intervals [CI]: 4.1–5.9), 5.2 (95% CI: 3.6–7.5), and 6.8 (95% CI: 4.6–10.1), respectively.

**Conclusions:**

Our findings strongly support the continued need to avoid beginning adult life as obese, as obese young men experience an increased morbidity, including fatal morbidity, from many diseases throughout life.

## Introduction

The prevalence of obesity has been increasing worldwide and in all age groups for some decades [1]. However, the impact of the epidemic on children and adolescents seems greater than in adults, reflected by relatively higher increases in overweight and obesity in the younger age groups [1], [2]. It is well known that obesity has detrimental health effects. A recent report by the Prospective Studies Collaboration, with data from 900,000 participants and 57 prospective studies, found that obesity is associated with an increased mortality from vascular diseases, diabetes, renal, hepatic, and respiratory diseases and cancer [3]. A common characteristic of these previous cohort studies is that the effect of obesity is investigated in predominantly middle-aged populations, which do not allow a distinction between recent or early development of obesity. In view of the still progressing epidemic of childhood obesity throughout the world [2], and the positive association between childhood obesity and obesity in young adulthood, it is important to get solid estimates of the health impact of this condition. Studies of the association between obesity in early adulthood and all-cause mortality has been conducted [4]–[7],and we have previously shown that men entering adult life as obese experience a doubling of mortality throughout life compared with the mortality in the underlying population [8]. Large-scale studies of the association between obesity in early adulthood and fatal morbidity has also been conducted [9]–[11]. However, the prospects of morbidity throughout life when starting adult life as obese are poorly elucidated. The aim of this study was to examine the association between obesity present in early adulthood and morbidity, assessed as incidence of first known event of disease, occurrence in the year preceding death, and prevalent disease at time of death and hence presumed to have contributed to the death.

## Methods

The study was conducted in accordance with Danish law for research based on individual data from registers, and permission was obtained from the Danish Data Protection Agency.

### Study participants

The present analyses are based on studies initiated in the early 1970's addressing obesity in the population of Danish young men examined at the draft boards since weighing was introduced in the examination [12]–[14]. This started in 1943 in District 1, the greater Copenhagen area, and in 1964 in District 2, the remainder of the Eastern part of Denmark. The young men were enforced by law to appear before the board at age 18 or shortly thereafter. Approximately 5% of the study population were exempted from examination as they were found unquestionable unfit for service due to illnesses or mental retardation. Obesity was not a condition allowing exemption [14]. Another 2% did not appear as they volunteered for service before the age of 18.

**Figure 1:**
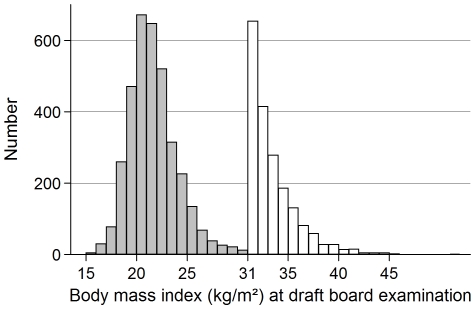
Histogram of the Distribution of Body Mass Index at Draft Board Examination for the 5,238 Men. Histograms illustrating the 100-fold enrichment of the right tail by the complete sampling of the obese cohort (BMI≥31.0).

Among the 362,200 Danish men examined at the boards in District 1 from 1943 through 1977 (no examinations took place in 1944 and spring of 1945, and no weighing in autumn of 1945 and 1946) and in District 2 from 1964 through 1977, the research group manually identified all obese men (n = 1,930) and a randomly selected control cohort of one in every hundred men (n = 3,601) in the archived paper files during the 1970s. Obesity was defined as 35% overweight relative to a Scandinavian standard in use at the time of sampling. This threshold later proved to correspond to a BMI≥31.0 kg/m^2^, which is above the 99.5^th^ percentile of BMI in this population (Figure 1). Thus, a study population was sampled, which represented the underlying BMI distribution of the population through the control sample, and where the right end of the distribution was 100-fold enriched relative to the controls.

### Body mass index

The medical personnel at the Danish military service measured the men's height without shoes and weight with only underwear. BMI was computed as weight in kilograms divided by square of height in meters.

### Covariates

*A priori*, draft board district and year of birth were identified as potential confounders. Draft board district is associated with obesity prevalence [13], and may also be associated with morbidity, because of both regional variation in morbidity and the different sampling periods in the two districts [14]. Year of birth is a potential confounder due to secular changes in disease occurrence combined with a rise in obesity prevalence in more recent time [13].

### Follow-up

All Danish residents alive on April 2, 1968 or born thereafter have been assigned a unique identification number in the Civil Registration System, which contain information on vital status [15]. Information on causes of death was obtained by linkage to the National Cause of Death Register, which was established on January 1, 1970 [16], [17]. Information on occurrence of disease was obtained by linkage to the National Hospital Discharge Register which was established on January 1, 1977 [17]. Follow-up of the men began at age at draft board examination or at their age in 1970 or 1977 (when the registers were established), whichever came later. Follow-up of the men ended at the date of death, loss to follow-up, emigration, or December 31, 2006 for analysis on the National Cause of Death Register, or on October 31, 2007 for analyses on the National Hospital Discharge Register.

### Analytical strategy

To keep the focus on men entering adult life as obese, only men between 18 and 25 years of age at draft board examination were included in the analyses. Consequently, 192 men not examined in this age range were excluded. One man could not be found in the registers, and further, 35 men died and 65 were censored before January 1, 1970, leaving 1,837 obese and 3,401 controls for the analyses of disease prevalent at time of death. For the analyses of incidence or occurrence of disease, 89 men died and 100 were censored before January 1, 1977, leaving 1,816 obese and 3,333 controls for these analyses. We also estimated the morbidity throughout life in the obese versus a normal weight reference group as defined by WHO (BMI: 18.5–24.9 kg/m^2^) [1].

The hospital discharge diagnoses and causes of death were classified according to the International Classification of Diseases, Eighth Revision (ICD-8) before 1994 and the Tenth Revision (ICD-10) thereafter. All the broad somatic disease categories were investigated, as well as sub-categories of well-known obesity-associated diseases as reported by WHO and the World Cancer Research Fund International [1], [18]. The incidence of diseases occurring after the draft board examination (or the establishment of the register) was analyzed in all men as time to first hospital admission following the draft board examination (or the establishment of the register) as recorded in the National Hospital Discharge Register. Further, we investigated the occurrence of disease in the year preceding death in men who died during follow-up. The reliability of the Cause of Death Register is based on how the certifying doctor fills in the death certificate. The diagnosis may be due to misclassification especially after Danish legislation has reduced the number of autopsies dramatically since 1990 [19]. Thus, investigating diseases occurring in the year prior to death could convey supplementary information on diseases that may have contributed to death. Finally, the series of diseases that had contributed to death according to the death certificate was investigated. In case of co-morbidity, an individual was only allowed to appear in each disease category once, but in several different disease categories simultaneously.

### Statistical methods

Data were analysed with Stata (version 9.2, Stata Corporation, TX). Cox proportional hazard regression was used to estimate hazard ratios (HR) of morbidity, with age as the underlying time axis and delayed entrance to ensure that the estimation procedures were based on comparisons of individuals of the same age. To take into account the putative confounding by draft board district we used stratified estimation allowing the baseline hazards to differ by district and sampling period; the stratification variable had four classes defined as District 1 with the examination periods split into 1943–1953, 1954–1963 and 1964–1977, and District 2 with the examination period 1964–1977. Year of birth was modelled as a continuous variable. To investigate whether the hazard of mortality changed across age the proportional hazard assumption was controlled visually by plotting the cumulative hazard at corresponding ages for the obese group versus the control cohort (or the normal weight reference group), a so-called double Nelson-Aalen plot [20]. Statistical interaction between the obese and the reference groups, and year of birth, respectively, was assessed by deviance tests based on comparisons of −2 log likelihood in nested models with and without cross-product terms.

## Results

Out of the 5,238 young men followed via the Civil Registration System 1,156 died during follow-up covering the age span from 18 through 80 years of age. Table 1 shows the characteristics for the obese and control cohorts. The majority of the men in the control cohort had a BMI within the normal range (18.5–24.9 kg/m^2^). The median year of birth was five years later for the obese than the controls, due to the increase in obesity prevalence in more recent time [13].

**Table 1:** Characteristics given as median (range) for the 1837 obese and 3401 controls.

		Obese cohort	Control cohort
BMI (kg/m^2^)		32.7 (31.0–51.8)	21.4 (15.7–30.9)
BMI, N*	Underweight	-	208
	Normal weight	-	2912
	Overweight	-	281
	Obese	1837	-
Year of birth		1951 (1918–1959)	1946 (1918–1959)
Age (yrs)†		19.0 (18.0–25.0)	19.0 (18.0–25.0)

Abbreviations: BMI: body mass index.

*As the control cohort was a random sample of the non-obese study population we show the contribution from underweight, normal weight and overweight men in this sample.

†age at draft board examination. The median age (range) for becoming at risk in the fatal morbidity analysis was 20.2 (18.3–51.5) for the obese and 23.6 (18.0–51.5) for the controls, and for the incidence analysis the corresponding ages were 25.3 (18.3–58.5) and 30.2 (18.3–58.5), respectively. This difference was due to the different timing in the establishment of the health registers.

**Figure 2:**
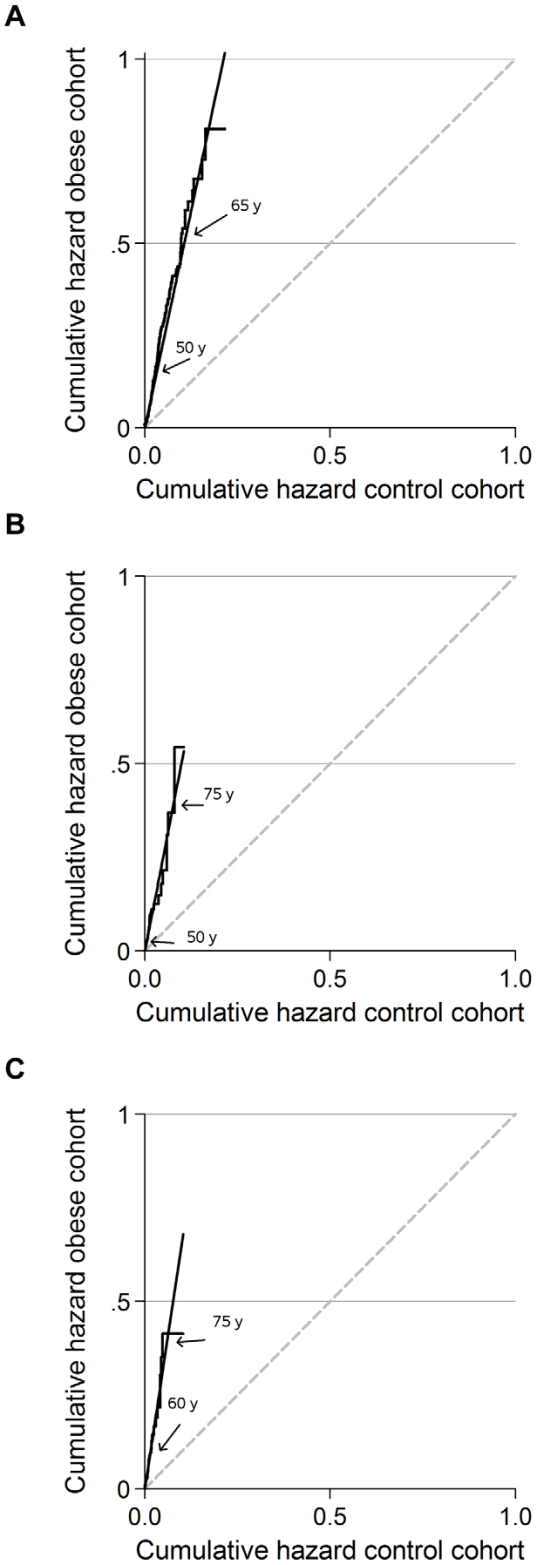
Cumulative hazard plot for the obese versus the control cohort at corresponding ages from 18 through 80 years. 2a. First incidence of diabetes. 2b. Occurrence of diabetes in the year preceding death. 2c. Prevalent diabetes at time of death. To investigate whether the hazard ratio changed across age, we created graphs of the cumulative hazard from the obese group versus the controls at corresponding ages. For every age the cumulative hazard in the obese group is plotted against the cumulative hazard in the control cohort. The arrows denote corresponding ages in the two cohorts. The grey, broken line is the line of equality. The interpretation of the black line is that the excess risk, measured on the hazard ratio scale, in the obese group is constant across the age range of observation from 18 through 80 years of age, and thus the assumption of proportionality in the Cox model is fulfilled.

Figure 2 illustrates the HR for first incidence of diabetes, diabetes occurring in the year prior to death, and prevalent diabetes at time of death, respectively, between the obese and control cohorts at equivalent ages. A curve concordant with the line of equality would show a constant HR of 1.00. The slope of the solid line illustrates the constant excess morbidity in the obese cohort, and equals the hazard ratio estimates, which were 4.9 (95% CI: 4.1–5.9), 5.2 (95% CI: 3.6–7.5), and 6.8 (95% CI: 4.6–10.1), respectively (table 2). The plots verify that the requirement of proportional hazards was fulfilled throughout the follow-up period. Similar plots were made for the remaining disease categories, and the proportional hazard assumption was verified for all except first incidence of IHD (see table 2).

**Table 2:** Disease-specific morbidity and mortality in obese men versus randomly selected Danish young men.

	Incidence of first known event				Occurrence one year prior to death				Prevalent at time of death			
	Controls (n)	Obese (n)	HR	95% CI	Controls (n)	Obese (n)	HR	95% CI	Controls (n)	Obese (n)	HR	95% CI
**Diseases of the circulatory system**	1,182	832	**2.1**	1.9–2.3	233	173	**3.3**	2.6–4.2	273	208	**3.3**	2.7 –4.1
Of which ischemic heart disease	419	317	**2.6** *	2.1–3.1	77	73	**4.7**	3.2–6.7	117	97	**3.8**	2.8–5.1
Of which cerebrovascular diseases	249	110	**1.4**	1.1–1.8	50	35	**3.9**	2.3–6.5	57	32	**2.8**	1.7–4.6
**Cancer**	457	155	1.2	1.0–1.5	210	69	**1.6**	1.2–2.1	221	76	**1.6**	1.2–2.1
Of which colorectal	72	25	1.5	0.9–2.4	24	10	**2.3**	1.0–5.2	26	7	1.4	0.6–3.4
Of which pancreas	13	8	**2.9**	1.1–7.5	11	7	**3.4**	1.2–9.3	10	8	**3.9**	1.4–10.7
Of which kidney	22	13	1.7	0.8–3.7	6	4	3.2	0.8–13.3	6	4	3.2	0.8–13.3
Of which oesophagus	16	8	1.5	0.6–3.9	10	6	2.2	0.7–6.7	10	4	1.5	0.4–5.3
**Endocrine, nutritional and metabolic diseases**	694	861	**3.3**	2.9–3.7	101	118	**4.8**	3.5–6.5	63	137	**7.9**	5.7–11.1
Of which type 2 diabetes	242	450	**4.9**	4.1–5.9	67	86	**5.2**	3.6–7.5	51	86	**6.8**	4.6–10.1
**Diseases of the digestive system**	1,084	601	**1.2**	1.1–1.4	115	73	**2.1**	1.5–3.0	92	64	**2.1**	1.5–3.0
Of which gallbladder disease	94	99	**2.4**	1.8–3.2	3	9	**9.0**	2.1–38.3	3	4	**5.4**	1.0–28.5
**Diseases of the respiratory system**	685	409	**1.4**	1.2–1.6	152	72	**2.2**	1.6–3.1	114	41	**1.6**	1.1–2.4
**Diseases of the musculoskeletal system/connective tissue**	1,057	679	**1.4**	1.3–1.5	38	41	**4.4**	2.6–7.4	6	7	**4.5**	1.2–16.4
**Diseases of the genitourinary system**	642	384	**1.6**	1.4–1.8	81	47	**2.9**	1.9–4.4	26	14	**3.1**	1.5–6.6
**Diseases of the nervous system**	414	283	**1.6**	1.3–1.9	62	26	1.6	0.9–2.7	34	12	1.1	0.5–2.3
**Infectious and parasitic diseases**	300	267	**2.0**	1.7–2.4	53	39	**2.5**	1.6–4.0	26	25	**2.6**	1.4–4.9
**Diseases of the blood (forming organs), immunol. disorders**	138	85	**1.7**	1.3–2.3	34	25	**3.6**	1.9–6.8	6	3	1.7	0.4–7.9
**Symptoms, signs, abnormal findings, ill-defined causes**	1,147	658	**1.3**	1.2–1.5	209	120	**2.2**	1.7–2.9	53	34	**2.2**	1.4–3.6

Abbreviations: n: number of events; HR: hazard ratio; CI: confidence interval.

All analyses are adjusted for year of birth and stratified by district and examination period.

Cause of death information was not available in the 35 (and 65 censored) men who died before January 1 1970 and the 69 men who died after December 31, 2006, respectively, and were therefore not included in the cause of death analyses.

A total of 91 men died (and 100 men were censored) before January 1 1977, and where therefore not included in the incidence analyses.

*The HR before the age of 60 years, hereafter it decreased to HR: 1.5 (95% CI: 1.0–2.3).

Overall, the obese had more than twice the mortality compared with the control cohort (HR: 2.1; 95% CI: 1.8–2.4). Further, for all major somatic disease categories the risk estimates for the obese were elevated and constant throughout adulthood, both when analyzed as incidence of first event after draft board examination (or the establishment of the register), diseases occurring in the year prior to death and disease prevalent at time of death (table 2). For diseases of the circulatory system the constant excess risk was illustrated by HRs of 2.1 (95% CI: 1.9–2.3), 3.3 (95% CI: 2.6–4.2) and 3.3 (95% CI: 2.7–4.1) for first incidence of disease, occurrence of disease in the year preceding death and prevalent disease at time of death, respectively. The similar risk estimates for cancer was somewhat increased in the obese group reflected by HRs of 1.2 (95% CI: 1.0–2.5), 1.6 (1.2–2.1) and 1.6 (1.2–2.1), respectively. Furthermore, the obese were at a significantly increased risk of becoming diseased by or dying from diseases of the respiratory system, diseases of the musculoskeletal system, diseases of the genitourinary system, infectious and parasitic diseases, as well as diseases of the blood. The risk of dying from, or with, diseases of the nervous system was also increased in the obese, although not significantly (table 2).

When using men with a BMI within the range of normal weight as reference essentially the same results were obtained (data not shown, may be obtained on request).

## Discussion

The present study shows strong associations between obesity in early adulthood and morbidity from most diseases assessed throughout adult life. Generally, the obese were more likely to become diseased earlier in life than the controls, and furthermore, the obese suffered from a constantly increased risk of dying from, or with, most diseases assessed.

With the obese cohort representing the most obese of all young men - above the 99.5^th^ percentile among 362,200 men - our study design provides great advantages for the analysis of this group. Conventional cohort studies usually lack sufficient unbiased series of obese individuals to get solid and valid estimates of the impact of this condition. Another strength of the present study was the very long follow-up, which allows detection of associations occurring both early and later in life. Further, in the time period investigated, examination for suitability for military service was mandatory (with few exemptions) leaving little suspicion of selection bias. We did not have information on smoking habits of the men. Smoking is a potential confounder and modifier of the investigated associations, e.g. smoking is associated with cardiovascular diseases that are likely to be increased among those who are overweight and obese [3], [21], [22]. A Swedish study of male draftees found no evidence of confounding or modification from smoking on BMI in relation to mortality [5]. Hence, using these results from a neighbouring population as basis, we consider our results as valid. Finally, one should be aware of potential detection bias when interpreting the results. An earlier detection of disease in the obese would result in inflated HRs for the obese relative to the controls, e.g. an obesity-related disease like diabetes may be affected by detection bias, because obese individuals are more likely to be tested for diabetes than lean individuals.

The report of a WHO consultation on obesity group the severe obesity-associated health problems into the four areas of cardiovascular problems, certain types of cancers, conditions associated with insulin resistance (e.g. diabetes), and gallbladder disease [1]. In accordance with the evidence gathered in the WHO report [1], diabetes and gallbladder disease showed the greatest HRs in this study, both when analyzed as incidence of first known disease event, occurrence in the year prior to death and as prevalence at time of death. The HR for diseases of the circulatory system was moderately increased, whereas the HR for cancer was slightly increased. Further, previous studies on young adults have investigated the association between increased BMI and ischemic heart disease (IHD). A Norwegian study of male and female adolescents between 14 to 19 years of age followed through middle age found that males with a BMI above the 85^th^ percentile (relative to the 25–74^th^ percentile) had a 2.9 increased risk of dying from IHD [9]. A Dutch study found a 2.5 times increased risk of IHD in 18-year-old men with a BMI greater than or equal to 25.0 (compared to the BMI reference category of 19.00–19.99) [10]. A study on Swedish draftees found a graded increase of IHD with increasing BMI; the obese (BMI greater than 30.0) had a four-fold increased risk of IHD compared to the reference category of BMI between 18.5 and 21.0 [11]. We found that the obese had a nearly four times increased risk of dying from, or with, IHD, relatively to the controls. Thus, we find the same pattern of effect for the well-known obesity-related diseases as previously reported, but can add to the current knowledge that the effect is present from early adulthood and is persistent throughout adult life.

In addition to the well-known obesity-related diseases discussed above, we also found elevated risk estimates in the obese for a broad range of other diseases, which included diseases of the respiratory system, diseases of the musculoskeletal system, diseases of the genitourinary system, diseases of the nervous system, infectious and parasitic diseases, as well as diseases of the blood. The risk estimates were elevated both when investigated as first incidence, occurrence in the year prior to death or as prevalence at time of death. Albeit with a different aim than the present study, a previous Swedish study on young men also described the negative effects of obesity in young adulthood [23]. They found that obese individuals were at an increased risk of disability pension from circulatory, musculoskeletal, and psychiatric diseases as well as diseases of the nervous system, tumors, injuries and other. Thus, our results are in line with results from a neighbouring country, showing that obesity in young adulthood is associated with a broad spectrum of diseases. However, the present study investigates a broader range of diseases than previously described, and in addition we investigate first incidence of disease as well as diseases occurring in the year before death and causes of death. Finally, the follow-up time of the present study covers adult life until 80 years of age, whereas the Swedish study only followed their individuals until middle age [23].

Finally, the risk estimates throughout the broad range of disease categories were constant throughout adult life. One could have expected that the obese men who at any given age have escaped the diseases in question, would have a declining risk of these diseases by advancing age if it was a sort of susceptibility to get the diseases that determines the age at onset. However, there was no decline with advancing age in the impact, measured on a HR scale, of obesity present when entering adulthood on a broad range of morbidities, including fatal morbidities. The study thereby provides an outlook for the rest of life for the obese when starting adult life with this condition. Whether this outlook may be altered by subsequent weight changes would be a relevant topic for further research.

Overall this study shows that obesity in early adulthood both increases the risk of becoming diseased earlier in life compared with the underlying population, as well as increases the risk of dying from a broad range of diseases. Notably these effects were constant, as measured on the HR scale, throughout adult life. Our results support the continued need to avoid beginning adult life as obese.
